# Beyond PD-1/PD-L1 Inhibition: What the Future Holds for Breast Cancer Immunotherapy

**DOI:** 10.3390/cancers11050628

**Published:** 2019-05-05

**Authors:** Sebastian Chrétien, Ioannis Zerdes, Jonas Bergh, Alexios Matikas, Theodoros Foukakis

**Affiliations:** Department of Oncology - Pathology, Karolinska Institutet, 171 76 Stockholm, Sweden; chretien.sebastian@gmail.com (S.C.); ioannis.zerdes@ki.se (I.Z.); Jonas.Bergh@ki.se (J.B.); alexios.matikas@ki.se (A.M.)

**Keywords:** breast cancer, checkpoint inhibitors, co-stimulatory, immunotherapy, novel targets, PD-1, PD-L1

## Abstract

Cancer immunotherapy has altered the management of human malignancies, improving outcomes in an expanding list of diseases. Breast cancer - presumably due to its perceived low immunogenicity - is a late addition to this list. Furthermore, most of the focus has been on the triple negative subtype because of its higher tumor mutational load and lymphocyte-enriched stroma, although emerging data show promise on the other breast cancer subtypes as well. To this point the clinical use of immunotherapy is limited to the inhibition of two immune checkpoints, Programmed Cell Death Protein 1 (PD-1) and Cytotoxic T-lymphocyte-associated Protein 4 (CTLA-4). Consistent with the complexity of the regulation of the tumor – host interactions and their lack of reliance on a single regulatory pathway, combinatory approaches have shown improved efficacy albeit at the cost of increased toxicity. Beyond those two checkpoints though, a large number of co-stimulatory or co-inhibitory molecules play major roles on tumor evasion from immunosurveillance. These molecules likely represent future targets of immunotherapy provided that the promise shown in early data is translated into improved patient survival in randomized trials. The biological role, prognostic and predictive implications regarding breast cancer and early clinical efforts on exploiting these immune-related therapeutic targets are herein reviewed.

## 1. Introduction

The recognition of the importance of the tumor – host interactions in the prognosis of cancer patients significantly predates the current era of cancer immunotherapy. The gradual deciphering of these complex interactions is summarized in the conceptual framework laid out by Hanahan and Weinberg [1], where immunoediting is suggested as a driving force guiding tumor progression. Exploiting these advances only in part, cancer treatment by the inhibition of negative regulators has revolutionized the management of multiple human malignancies, culminating with the award of the 2018 Nobel Prize in Physiology or Medicine – the first ever bestowed upon research related to an anticancer therapy [2]. 

Beyond its utility as a treatment target, immune response to cancer has also been a subject of research concerning its role as both a prognostic and predictive biomarker. As an example, higher tumor-infiltrating lymphocyte (TIL) counts and expression of immune function genes have been shown to predict better outcomes in most breast cancer (BC) subtypes and increased rates of pathologic complete remission (pCR) following the administration of neoadjuvant chemotherapy (NACT) for early BC (EBC) [3,4]. In metastatic BC (MBC), TIL enumeration has not proven to be as successful [5], since TIL counts have been shown to be lower in metastatic sites compared to the primary tumor [6]. On the other hand, Programmed Cell Death Protein 1 (PD-1) and its ligand PD-L1, as well as cytotoxic T-lymphocyte-associated protein 4 (CTLA-4), have been extensively evaluated as putative markers of response to immunotherapy with PD-1/PD-L1 and CTLA-4 blockade, respectively [7,8]. Although data stemming from randomized clinical trials in various human cancers are conflicting, in MBC only one phase 3 trial has been reported demonstrating increased benefit from the combination of atezolizumab and nab-paclitaxel compared to nab-paclitaxel alone in patients whose tumors expressed PD-L1 [9]. PD-1/PD-L1 and CTLA-4 checkpoint inhibitors are already the focus of advanced clinical trials (reviewed by Adams et al. [10]). 

**Figure 1:**
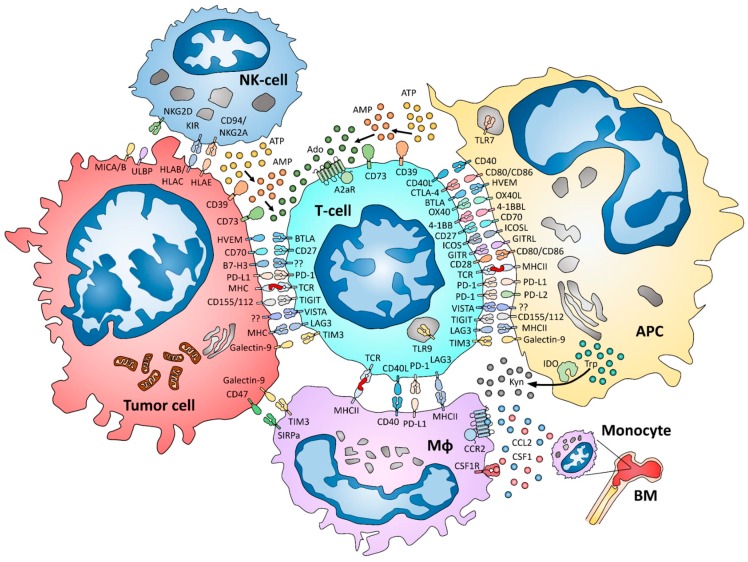
Interplay between tumor cells and immune system components in the tumor microenvironment. Abbreviations for represented cells and immune-related markers are explained in the main text.

Despite the aforementioned exciting developments, it is clear that only a fraction of the potential immunologic therapeutic targets has been comprehensively characterized. Unfortunately, research on immunotherapy for BC has lagged behind due to its perceived lower immunogenicity [11]. Nevertheless, a growing body of literature focusing on a large number of co-stimulatory and inhibitory molecules suggests that the field of cancer immunotherapy in general, and BC in particular, is only in its early stages of development. Herein, we summarize available data on novel immunotherapy targets with a focus on BC (Figure 1). 

## 2. Markers Predominantly Expressed on T-lymphocytes 

### 2.1. LAG-3

Lymphocyte activation gene-3 (LAG-3) is a cluster of differentiation 4 (CD4) related negative regulator of immune response considered as a marker of T-cell exhaustion. It is expressed on both effector and regulatory T-cells, Natural Killer (NK)-cells, B-cells and dendritic cells (DC) [12,13,14,15,16]. Identified LAG-3 ligands are MHC (Major Histocompatibility Complex) class II molecules expressed on antigen presenting cells (APC), LSECTin and Galectin-3 [17,18]. LAG-3 is thought to inhibit the activity and expansion of effector T-cells and enhances the suppressive activity of T-regulatory lymphocytes (Tregs) [19,20,21,22]. 

Published data on the role of LAG-3 in BC indicate that it is overexpressed in the tumor compared to the adjacent healthy breast tissue [23,24,25], while its overexpression has been associated with improved patient outcomes [26] (Table 1). Following promising pre-clinical results, LAG-3 inhibitors are currently being tested in early phase clinical trials including BC, as monotherapy or in combination with chemotherapy or anti-PD-1 therapy (Table 2). One phase I/II clinical trial testing IMP321 (Eftilagimod), a recombinant soluble LAG-3 Ig (Immunoglobulin) fusion protein, in combination with weekly paclitaxel as a first line treatment in 30 patients with MBC showed promising results, with a response rate of 50% [27]. 

### 2.2. TIM-3

T-cell immunoglobulin and mucin domain-containing protein 3 (TIM-3) is a negative regulator of adaptive and innate immune responses. It is expressed on CD8+ and CD4+ T helper 1 cells (Th1 cells), Tregs, NK cells, DC, monocytes and macrophages [89,90,91,92]. Known ligands to TIM-3 are Galectin-9, Ceacam1, HMGB1 (High Mobility Group Box 1) and phosphatidylserine, all expressed by a variety of cells including tumor cells [93,94,95,96]. TIM-3 induces an immunosuppressive environment by suppressing effector Th1 response [93], regulating CD8+ T cell exhaustion [97] and enhancing the regulating function of Tregs [90,98]. It also inhibits the stimulation of the innate immune response by competing with tumor-derived nucleic acids to bind HMGB1 and promoting the expansion of myeloid-derived suppressor cells (MDSC) [95,99].

TIM-3 seems to be upregulated both in BC samples compared to normal adjacent tissue and circulating lymphocytes, possibly through hypomethylation of its promoter [23,29] (Table 1). However, expression on immune cells has been reported to vary widely [29,100]. Burugu et al. evaluated TIM-3 IHC expression in 3992 BC samples of all subtypes and found that the TIM-3 intraepithelial TIL infiltration is associated with a better outcome [32]. TIM-3 polymorphisms might also play a role in the susceptibility to, and prognosis of BC [101,102,103]. 

Drugs targeting TIM-3 are currently being tested in early phase clinical trials including BC, alone or in combination with anti-PD1/PD-L1 check point inhibitors, with no published results yet (Table 2).

### 2.3. VISTA

V-domain Ig suppressor of T cell activation (VISTA) is a negative regulator of the T-cell immune activity functioning both as a ligand and receptor [104]. It has been shown to be expressed by CD4+ and CD8+ T-cells, Tregs, DC, NK-cells, monocytes, macrophages and granulocytes [105,106], as well as tumor cells [107,108,109]. VISTA exerts its immunosuppressive function by decreasing the T-cell production of effector cytokines, diminishing T-cell proliferation and increasing conversion to Tregs [106]. To our knowledge, VISTA’s expression and prognostic impact in BC has never been assessed, although a phase 1 clinical trial which enrolls TNBC patients and tests an oral inhibitor of PD-L1, PD-L2 and VISTA is currently ongoing (Table 2).

### 2.4. TIGIT

T-cell immunoreceptor with Ig and ITIM domains (TIGIT) is a co-inhibitory molecule expressed on effector, memory and regulatory T-cells, follicular helper (Tfh) and NK-cells [110,111]. It competes with CD223 to bind its two identified ligands, CD155 and CD112, expressed on APC, fibroblasts, endothelial, epithelial cells and also on a variety of cancer cells, including BC [112]. TIGIT has different ways of exerting its immunosuppressive action: Direct inhibition of NK-cell function [113], direct inhibition of T-cell activation, proliferation and cytotoxicity by attenuating TCR-driven (T-cell receptor) activation signals [114] and indirect inhibition of T-cells by promoting the maturation of immunoregulatory DCs [111]. It also promotes the Tregs function by being a direct target to FoxP3 (Forkhead box P3) and inducing an enhanced suppressive function [115,116].

TIGIT expression in BC has only been assessed at the transcriptomic level, with most studies showing overexpression [23,31,33,117] (Table 1). In one study, overexpression was correlated with improved patient survival in TNBC [33], leading to the development of antibodies targeting TIGIT in combination with PD-1 blockade (Table 2). 

### 2.5. GITR

Glucocorticoid-induced TNFR-related protein (GITR) is a co-stimulatory member of the tumor necrosis factor (TNF) receptor superfamily expressed constitutively on all T-cells [118,119]. It is also expressed on NK-cells, eosinophils, basophils, macrophages and B-cells [120]. Its activating ligand is the GITR ligand (GITRL), expressed on APC and endothelial cells [121,122]. Upon binding, GITR exerts an immunostimulatory activity by directly enhancing T-cell proliferation and effector functions [123,124]. It also indirectly enhances the effector T-cell function by decreasing the intratumoral Treg numbers and suppressive function [125,126]. By avoiding activation-induced cell death, it also promotes an increase in memory T-cells [127]. 

Cari et al. assessed GITR mRNA expression in 3169 BC patients of all subtypes and found an overexpression in 42% of the cases [31]. Other studies demonstrated that expression is increased in both infiltrating [34] and circulating Tregs of BC patients [35,37]. Interestingly, GITR seems to also be overexpressed in CD4+ T-cells in BC-infiltrated lymph nodes [36] (Table 1). 

BMS-986156, a GITR agonistic monoclonal antibody, in combination with nivolumab has demonstrated an acceptable safety profile and promising antitumor activity in advanced solid tumors [82]. Other agonist molecules targeting GITR are currently being tested in early phase clinical trials (Table 2).

### 2.6. B7-H3

B7 homolog 3 (B7-H3) is a member of the B7 family of immunomodulatory ligands. It is not spontaneously expressed in peripheral blood mononuclear cells but can be induced upon stimulation in APC, T-cells and NK-cells [128]. It is widely expressed in healthy solid organs and several malignancies, including BC [129]. Interestingly, it is also expressed by tumor-associated endothelial cells [45]. Although B7-H3 was initially seen as a co-stimulatory molecule, which increases CD4+ and CD8+ proliferation and enhances T cell cytotoxicity [129,130], the majority of recent studies highlight its co-inhibitory role. Indeed, it appears to downregulate T-cell proliferation and cytokine production [131], Th1 and Th2-mediated immune reactions [132] and inhibit NK cells activity [133]. Moreover, B7-H3 seems to influence cancer progression beyond its immunoregulatory role, by promoting migration, invasion and angiogenesis [134,135].

B7-H3 expression in BC has been extensively studied and demonstrated to confer worse prognosis [41,42] (Table 1). As a result, two antagonist drugs – a monoclonal antibody (enoblituzumab) and a dual-affinity re-targeting (DART^®^) protein (MGD009) – are currently under evaluation in early phase clinical trials including BC (Table 2).

### 2.7. ICOS

Inducible T cell co-stimulator (ICOS) is a specific T-cell molecule of the B7-binding CD28 family, expressed on activated T-cells after TCR engagement and enhanced by CD28 co-stimulation [136,137]. Its only ligand is ICOS-L, mainly expressed on APC [138,139,140] but also on endothelial and lung epithelial cells [141,142]. Although typically seen as an immune co-stimulatory pathway, notably through promoting cell proliferation/differentiation, enhancing Th1/Th2 function and facilitating T-dependent B-cell activation [136,137,143], ICOS/ICOS-L interaction might also have an immunosuppressive role through the accumulation of Tregs and secretion of IL-10 [46,144].

In a study by Faget et al., BC patients overexpressing ICOS had a significantly worse survival in the univariate but not multivariate analysis [46], while certain ICOS gene polymorphisms have also been associated with increased BC susceptibility in Chinese populations [145,146] (Table 1). Ongoing trials of agents targeting ICOS are shown in Table 2. 

### 2.8. 4-1BB (CD137)

4-1BB (CD137) is a member of the TNF receptor superfamily, widely expressed on adaptive and innate immune cells like effector, helper and regulatory T-cells [147,148], B-cells [149], NK-cells [150,151], DCs [152], neutrophils, eosinophils, mast cells, monocytes and macrophages [153]. It is also expressed by a variety of other non-immunological cells, including endothelial and malignant hematological cells [154]. It exerts a co-stimulatory action upon ligation with its ligand 4-1BBL, resulting in enhanced T-cell and NK-cell proliferations, production of pro-inflammatory cytokines and cytotoxicity [150,155,156] and the inhibition of activation-induced cell-death in T-cells [157]. 

Two studies using gene-expression datasets demonstrated that 4-1BB is overexpressed in BC and is associated with better prognosis [31,47] (Table 1). 

Monoclonal agonist antibodies are currently being tested in early phase clinical trials including BC (Table 2). Two early-phase studies (NCT00351325 and NCT00309023) raised concerns due to two hepatotoxicity-related deaths, though not replicated in a follow-up phase 1 study [158].

### 2.9. CD27 and CD70

CD27 and its only ligand CD70, are members of the TNF receptor and ligand superfamily that interact exclusively with each other. CD27 expression on T-cells is tightly regulated, with upregulation upon activation after the TCR stimulation followed by downregulation once the effector T-cell differentiation is acquired [159]. CD27 is also expressed on B-cells (germinal center and memory B-cells) and NK-cells [160,161,162]. CD70 expression on immune cells is also tightly regulated and is present on activated T-cells, stimulated B-cells, mature DC and NK-cells [163,164,165,166]. Interestingly, CD70 has also been found to be expressed in various hematological, sarcoma and carcinoma cells including BC [167]. The CD27-CD70 pathway exerts its co-stimulatory activity in great part through CD27 interaction with TNF receptor associated factors (TRAF), resulting in the activation of transcription factors of MAPK (Mitogen-activated Protein Kinase) and NFκB (Nuclear Factor kappa-light-chain-enhancer of activated B-cells) family. This leads to the expansion and survival of activated T cells [168,169,170,171,172,173]; differentiation to memory and effector T-cells [173,174,175]; activation of NK-cells [176,177]; and differentiation plus activation of B-cells [178,179,180].

CD70 protein expression in BC was assessed in two studies with contrasting results [49,50] (Table 1). Of interest, Liu et al. demonstrated that a high CD70 expression was correlated with worse lung metastasis-free survival, but not with other metastatic sites following relapse of EBC. In addition, gene expression studies showed that CD70 was overexpressed in basal-like compared to Luminal A cancers and that overexpression after NACT was associated with a better outcome [51,181]. 

Two antibodies, ARGX-110 targeting CD70 and CDX-1127 (Varlilumab) targeting CD27 are currently in early phase clinical trials. In addition, a trial is testing the safety and activity of administering peripheral blood lymphocytes transduced with a CD70-binding Chimeric Antigen Receptor (CAR) to patients with CD70-expressing cancers (Table 2).

### 2.10. OX40 and OX40L

OX40 (CD134) and OX40L are members of the TNF superfamily. OX40 is constitutively expressed on Tregs and transiently induced on activated CD4+ and CD8+ T-cells following TCR stimulation [182,183,184]. It has also been reported to be expressed by neutrophils, NK-cells and NKT-cells [185,186,187]. Its ligand, OX40L, is expressed on professional APC, NK-cells, Langerhans cells, vascular endothelial cells, monocytes, neutrophils and mast cells. Like OX40, it is upregulated upon activation [188,189,190,191,192,193,194,195]. OX40-OX40L interaction, like other TNF members, exerts a co-stimulatory effect through interacting with TRAF, which impacts CD4+ and CD8+ T cells by enhancing their proliferation and survival, generating memory cells, enhancing their effector function and promoting differentiation into Th1, Th2 and Th17 cells through various cytokines production [196,197,198,199,200,201,202,203]. 

Several studies have assessed OX40 expression in BC, showing an expression varying from 15.5% to 85% of cases (Table 1). Interestingly, Xie et al. reported expression on cancer cells while all the other studies reported expression on TILs [52]. Consequently, a number of agonistic monoclonal antibodies targeting OX40 and a mRNA encoding OX40L (injected intra-tumorally) are currently being tested in early phase clinical trials including BC, alone or in combination with other immunotherapies. (Table 2)

### 2.11. BTLA

BTLA (B and T Lymphocyte Attenuator) is an inhibitory Ig-domain-containing glycoprotein receptor of the CD28 superfamily expressed on activated T-cells, B-cells, Tfh cells, macrophages, DC, NKT-cells and NK-cells [204,205,206,207,208]. Its only proven ligand is HVEM (Herpes Virus Enter Mediator), a member of the TNF receptor family, expressed on CD4+ and CD8+ T-cells (strongly on resting T cells, downregulated upon activation), naïve and memory but not activated B-cells, monocytes, DC, solid organs, tumor-associated endothelial cells or on various cancer cells including BC [209,210,211,212]. BTLA has also been described as a potential receptor for B7-H4 in BC [213]. BTLA exerts its T-cell inhibitory action upon binding HVEM, leading to a decreased T-cell proliferation and cytokine production with a predominant effect on CD4+ cells [214,215,216,217,218,219]. Data concerning its action on B-cell function is scarce but it appears to negatively regulate B-cell activation [220]. Interestingly, BTLA and PD-1 seem to be co-expressed on CD8+ T-cells. 

Data concerning BTLA expression in BC is scarce (Table 1). Although it seems to be overexpressed at the transcriptomic level, especially in TNBC where it was also associated with improved survival [57], protein expression appeared to be limited in another study [58]. To our knowledge, no clinical trials for therapeutic targeting of BTLA are currently ongoing.

### 2.12. TLR9

Toll-like receptors (TLRs) are type I transmembrane glycoproteins of the pattern recognition receptors (PRR). They play a key role in immunity by allowing immune cells to recognize non-self or altered-self molecular patterns, activating the innate immune response and coordinating the innate and adaptive immune responses. The most studied member in BC is the intracellular receptor TLR9.

TLR9 is a DNA receptor that migrates from the endoplasmic reticulum to the endosomal/lysosomal compartment when DNA enters the cell [221,222]. When activated by DNA recognition, TLR9 initiates a signaling cascade [222,223], leading to the activation of various transcription factors like NF-κB and AP-1 (Activator protein 1) [224], thus promoting the transcription of genes that are important for inflammatory and immune responses [225,226]. In addition, it promotes adaptive immunity by enhancing DC maturation and producing a favorable cytokine/chemokine milieu that results in the activation of Th1 and CD8 cytotoxic T lymphocytes as well as by promoting B-cell proliferation [227,228].

TLR9 expression and its prognostic role in BC has been reported by several studies with conflicting results [60,64] (Table 1). Nevertheless, it appears that TLR9 is expressed at higher levels in estrogen receptor (ER) negative and high-grade tumors. Regarding the prognostic significance of TLR9 expression, three studies associated high expression with a better outcome [60,61,64], while two other studies reported worse survival [63,66]. Of interest, Karki et al. demonstrated that BC patients have decreased serum levels of TLR9 compared to patients with benign lesions and healthy controls, proposing it as a potential diagnostic biomarker [229]. Moreover, several but not all studies have shown an association between TLR9 gene polymorphisms and BC susceptibility [230,231,232,233].

Therapeutic targeting of TLR9 has proven to be efficient in pre-clinical models of various cancers including BC and many drugs are currently being tested in several cancer types, some of them even reaching phase III (NCT03445533) (Table 2). 

### 2.13. The Adenosine Pathway in Breast Cancer

The adenosine pathway is an important peripheral control mechanism for regulating the immune response in order to prevent over-activation and tissue damage. As with other immunoregulatory pathways, cancer cells are capable of hijacking it in order to promote tumor escape. Important components of this pathway are the adenosine receptor A2a (A2aR), through which the extracellular adenosine can activate its intracellular signaling pathway and the ectonucleotidases CD39 and CD73, which participate in extracellular adenosine production by dephosphorylating ATP.

A2aR is a G-protein-coupled receptor expressed on T and NKT-cells, B-cells, monocytes, macrophages, DC, NK-cells, mast cells, eosinophils and platelets [234]. CD73 is a cell-surface enzyme that can also be found as an enzymatically active soluble form. It is widely expressed on immune cells including B-cells, CD8+ and CD4+ T-cells, Tregs, neutrophils, MDSC, monocytes, macrophages, DC and NK-cells [235]. It is also expressed on a wide range of epithelial cells, endothelial cells and cancer cells including BC [235,236,237]. CD39, another cell-surface enzyme which produces adenosine, is also expressed on a variety of immune cells [238,239,240]. It is also expressed on platelets, endothelial cells and cancer cells including lung, melanoma, pancreatic and lymphoma cells [241,242,243]. Like CD73, a soluble catalytically active form of CD39 exists [244]

The adenosine pathway exerts an immunosuppressive action by inhibiting effector T-cell activation [245], proliferation, cytokine production and cytotoxicity as well as promoting their immunosuppressive cytokine production [246,247]. In addition, it promotes Tregs formation [246], inhibits NK-cell antitumor activity [248], NKT-cell production of cytokines [249], macrophage proliferation [250] and DC maturation [251]. It has also been shown to increase the expression of other immune checkpoints [252]. 

CD73 expression on BC cells ranges from 9 to 84% of the cases and is generally associated with worse outcome, although one study reported contrasting results [68] (Table 1). In addition, CD39 is overexpressed both in TILs and circulating T cells of BC patients when compared to healthy controls, but its prognostic value has not been studied.

Numerous pre-clinical studies have demonstrated the efficacy of targeting the adenosine pathway in BC models, leading to the development of A2aR oral inhibitors and antibodies targeting CD73, currently in early phase clinical trials (Table 2). CD39 targeting therapies are currently under pre-clinical development but to our knowledge none have yet reached clinical trials.

## 3. Tumor-Associated Macrophages and Related Markers

Tumor-associated macrophages (TAMs) represent a major and heterogeneous distinct immune cell subpopulation in the tumor microenvironment (TME). In many tumor types, including BC, TAMs play a key role in tumor progression, angiogenesis, immune evasion and metastasis [253]. They also interact with other cell types through the secretion of various cytokines which in turn can modify the balance between tumor, stromal, endothelial and immune cells. According to the markers expressed on their cell surface as well as the factors they secrete, TAMs can be divided into two subtypes: a) the classically activated M1-like macrophages which have pro-inflammatory, anti-tumoral properties mainly through the secretion of TNF-a (Tissue Necrosis Factor alpha), IL-1, IL-2, IL-6, IL-12; and b) the selectively activated M2-like macrophages with anti-inflammatory, pro-tumoral phenotype mainly through TGF-β (Transforming growth factor beta), IL-4, IL-10 and IL-13 [254]. In terms of prognosis, TAMs were associated with worse overall survival in many solid tumors according to a large meta-analysis [255]. In BC in particular, a meta-analysis of sixteen studies revealed that a high TAM density was associated with worse overall survival (Hazard Ratio [HR]=1.50; 95% Confidence Intervals [CI] 1.20-1.88) and disease-free survival (HR=2.22; 95% CI 1.71-2.89) [256]. Overall, therapeutic strategies against TAMs are based on two major approaches: a) targeting TAM recruitment and activation, and b) reprogramming macrophage polarization towards an anti-tumoral phenotype. The first approach includes the elimination of TAM and monocyte accrual to the tumor site through the inhibition of mainly CSF-1/CSF-1R (Colony Stimulating Factor 1/ Colony Stimulating Factor 1 Receptor) and CCL2/CCR2 (C-C Motif Chemokine Ligand 2/ C-C Motif Chemokine Receptor 2) signaling axes. The second approach relies on the fact that TAMs are mostly of the M2-like phenotype and thus, stimulating the properties of the M1-like phenotype could be an effective treatment option to restore anti-tumoral activity. Such potential treatments for the macrophage polarization shift include CD40-agonists and/or TLR7 agonists. Whether the aforementioned therapeutic agents can be combined with other therapies which can target angiogenesis, increase phagocytic activity or enhance anti-tumor immunity is currently under investigation [257,258]. Moreover, recognition and targeting of other pro-tumoral chemokines and cytokines [259] or novel targets could broaden the therapeutic spectrum in cancer immunotherapy. 

### 3.1. CSF-1/CSF-1R

TAM recruitment is highly controlled by the interaction of the glycoprotein CSF-1 with its receptor CSF-1R, a member of type III receptor tyrosine kinase family. Binding of CSF-1 to CSF-1R leads to activation, recruitment and proliferation of TAMs [260]. CSF-1R is normally expressed in various cell types but its expression in BC cells has been correlated to worse prognosis [261,262,263,264] (Table 3). Therapeutic targeting of this axis is under active investigation (Table 4). 

### 3.2. CCR2/CCL2

The recruitment of circulating monocytes from the bone marrow into the TME is also mediated by the expression of the chemokine ligand CCL2. The binding to its receptor CCR2 leads to the differentiation of monocytes into TAMs and to the subsequent promotion of their pro-tumoral activity, tumor cell proliferation, angiogenesis and metastatic dissemination [265,266]. Expression of these chemo-attractants has been linked to worse prognosis in BC patients [267,268,269,270,271] (Table 3). Targeting this axis using CCR2 antagonists and anti-CCL2 antibodies is currently being explored in advanced solid malignancies, including BC (Table 4).

### 3.3. CD47 and SIRPa

Interaction between the two cell-surface immunoglobulin family members, CD47 and signal regulatory protein alpha (SIRPα), is crucial for the regulation of phagocytosis. CD47 is expressed on cancer cells while SIRPα is expressed on macrophages. Upon interaction, the anti-tumor immunity is diminished as CD47 represents a ‘don’t eat me’ signal, thus impairing phagocytosis [296,297]. Through targeting this checkpoint axis using anti-CD47 antibodies, CD47-Fc and/or SIRPα-Fc fusion proteins, the macrophage phagocytic capacity can be restored (antibody-dependent cellular phagocytosis, ADCP) towards an effective immune response. The first reported efficacy results of the Hu5F9-G4 inhibitor combined with rituximab in non-Hodgkin’s lymphoma are promising [298]. Possible synergistic effects of such treatments with anti-HER2 or anti-PD-L1/PD-1 antibodies are being tested in clinical trials (Table 4).

### 3.4. TLR7

TLR7 represents an intracellular receptor, member of the toll-like receptors transmembrane glycoprotein family. Its expression can enhance the DC function and can re-programme macrophages towards an anti-tumoral M1 phenotype [299,300]. Therefore, its activation using TLR7 agonists could provide effective anti-tumor responses. Indeed, the use of the topical TLR7-agonist imiquimod in combination with nab-paclitaxel led to the short-term regression of BC cutaneous metastases in early phase trials [301,302] (Table 4). 

### 3.5. CD40

CD40 represents a co-stimulatory protein, member of the TNF receptor family and is an emerging target in cancer immunotherapy. CD40 is mostly expressed by APC and macrophages and binding of its ligand (CD40L) on T-cells results in T-cell activation [303]. Preclinical data of the CD40-agonist efficacy have been reported in BC and other tumor types, demonstrating the promotion of T-cell responses [304,305]. CD40 activation using agonistic monoclonal antibodies can also lead to the enhancement of macrophage tumoricidal and pro-inflammatory properties mainly through MHC-II upregulation [303]. Preliminary results indicate activity and durable immune responses [306] (Table 4).

## 4. Natural-Killer Cells and Related Markers

### 4.1. Killer Immunoglobin Receptors (KIR)

NK-cells represent an immune cell subpopulation with an active role in effective antitumor immunity [307]. MHC class I specific Killer Immunoglobin Receptor (KIR) family members are mostly expressed on the surface of NK-cells. Some KIR - upon binding to their ligands HLA-B or HLA-C - can hinder NK cell activation [308], while others are associated with NK stimulatory properties and better prognosis for cancer patients [309,310]. Ongoing clinical trials are underway, testing antibodies against NK-inhibiting KIR family members in combination with other immune checkpoint inhibitors (Table 4).

### 4.2. CD94/NKG2A

NK group member 2A (NKG2A) represents a novel inhibitory receptor, which forms heterodimers with CD94, both belonging to the C-type lectin-like family and expressed mainly on the surface of NK-cells and also on CD8+ T-cells. Upon binding of the complex to its MHC class I (HLA-E) ligand, the anti-tumoral capacity of NK-cells can be hindered and an immunosuppressive phenotype through T-cell inactivation is established [308,311]. Recently, two preclinical studies in colorectal and head and neck carcinoma demonstrated that blockade of this receptor may be a new appealing immunotherapeutic target [312,313]. Expression of NKG2A has been described in BC patients [273], however no studies on therapeutic targeting are ongoing (Table 3).

### 4.3. NK-Cell Activating Receptors

NK-cells are activated through various receptors such as the natural cytotoxicity receptor (NCR) family (NCR1 or NKp46, NCR2 or NKp44, NCR3 or NKp30) and NK group member 2D (NKG2D). The latter recognizes several ligands including MHC class I polypeptide-related sequence (MICA/MICB) and UL16-binding proteins (ULBP1-6) and their interaction leads to enhanced cytolysis [314,315]. Expression of NKG2D ligands has been associated with improved survival in BC [274,316,317] (Table 3). 

## 5. IDO

Indoleamine 2,3 dioxygenase-1 (IDO1) is an enzyme mostly found in DC and an appealing target for cancer immunotherapy [318]. It plays an important role in metabolism-mediated immune regulation by catalyzing the conversion of amino acid tryptophan to kynurenine and thus impairing T-cell activation and promoting Treg expansion [319,320]. IDO expression in BC patients has been extensively studied, with varying positivity, from 14 to 100% of the cases [276,279]. Most of the studies describe a predominant expression by tumor cells with limited expression by stromal dendritic-like cells and occasional expression by myoepithelial cells. Although conflicting results have been reported, the majority of the studies show that the IDO expression is correlated to an advanced stage at diagnosis, high grade, ER negativity and worse outcome [277,278]. Recent findings from a phase I trial, indicate the activity and safety of targeting IDO in combination with anti-PD-L1 monoclonal antibody atezolizumab in various advanced solid tumors including BC [321].

## 6. Myeloid-Derived Suppressor Cells

MDSCs represent a heterogeneous population of immature myeloid cells including progenitor cells, immature DCs, macrophages and granulocytes. In humans, MDSCs are defined by the positive expression of CD33 and CD11b and negative or reduced expression of HLA-DR. MDSCs are further classified as monocytic or granulocytic MDSCs when CD14 or CD15 is expressed, respectively. 

MDSCs play a major role in promoting an immunosuppressive microenvironnment through various mechanisms: Production of reactive oxygen and nitrogen species depleting TILs [322,323], impairment of lymphocyte-homing [324], promotion of other immunosuppressive cells such as Tregs and M2-macrophages [325,326], depletion of metabolites involved in the T cell function such as L-arginine and cysteine [327,328] PD-L1 expression [329] and adenosine production by upregulating the expression of ectonucleosidases CD39 and CD73 [330]. In addition to their immunosuppressive effect, MDSCs also promote tumor dissemination and metastasis by affecting epithelial-mesenchymal transition [331], degradation of extra-cellular matrix [332], stem cell formation [333], angiogenesis and formation of premetastatic niches [334,335]. 

Presence of MDSCs in BC patients has been studied both in peripheral blood and primary tumors. Patients with BC have elevated levels of circulating MDSCs compared to healthy donors or patients with benign lesions and the levels of MDSCs increase with tumor burden (i.e. clinical stage), making it a potential tool for BC diagnosis [336,337]. MDSCs are also present in the BC tumor microenvironment at significantly higher levels than the adjacent healthy breast tissue and one study found that TNBC seems to be more infiltrated than other BC subtypes [338,339,340]. Moreover, MDSCs represent a potential biomarker for predicting both survival and response to NACT, with higher levels of circulating of infiltrating MDSCs being associated with worse survival and pCR rates [340,341,342,343].

As a result, targeting MDSCs is a putative therapeutic tool for BC patients and different strategies have shown promising results in pre-clinical studies [344,345,346,347]. Briefly, current treatment strategies aim to modulate myelopoïesis by forcing differentiation into mature cells or inhibiting maturation from precursor cells, block MDSC accumulation in tumor sites and block MDSC immunosuppressive functions [348]. To our knowledge, only pre-clinical data of MDSC targeting in BC have been published but three early-phase clinical trials are currently ongoing (NCT03145012; NCT02922764; NCT02499328).

## 7. Implementing Combination Immunotherapy in the Clinic

Blockade of the PD-1/PD-L1 axis through the use of monoclonal antibodies as monotherapies has met with considerable success during the past decade. The central concept of immunotherapy with the inhibition of negative regulators of the immune response is the restoration of activity of exhausted cytotoxic T-lymphocytes. As evidenced by the observation of responses among patients lacking a local immune response (no PD-1/PD-L1 expression at the protein level, absence of TIL), a pre-existing immune response is not an absolute prerequisite needed for the elicitation of responses to treatment. Nevertheless, response rates and response duration following treatment with a monotherapy seem to be lower among those patients [349]. 

Intriguingly, the combined immune checkpoint blockade confers superior results compared to PD-1 blockade alone in this patient group. Data derived from the phase 3 CheckMate 067 trial indicate that double PD-1 and CTLA-4 blockade with nivolumab and ipilimumab improved both progression-free (HR=0.67; 95% CI were not reported) and overall survival (HR = 0.70) compared with nivolumab alone in patients with metastatic melanoma and PD-L1 expression lower than 1% [350]. Although this analysis is exploratory and the trial was not designed to perform this comparison, it provides support for immunotherapy combinations. The theoretical background seems intuitive. Mechanistically the two checkpoints function on different sites of immune activation: CTLA-4 carries out its function at the sites of priming whereas PD-1 is responsible for maintaining tolerance by dampening the activation of T-lymphocytes in the periphery [351]. It is unclear however whether the combinatory approach is successful thanks to an additive effect of the two inhibitors or if it results from the suppression of escape mechanisms. Similarly, it is conceivable that the inhibition of other negative regulators or agonistic activation of co-stimulatory molecules in combination with each other or with established immunotherapies can lead to further improvements in terms of patient outcomes. It is clear however that a mechanistic understanding of the biology of the candidate therapeutic targets and of the cross-talk that is activated upon inhibition is of paramount importance. Further underscoring the need for a deep understanding of the underlying biologic processes and the rational design of novel agents is the failure of the combination of the once promising IDO1 inhibitor epacadostat to improve outcomes in combination with pembrolizumab versus pembrolizumab alone in patients with metastatic melanoma [352]. 

While increased efficacy is the main goal, two barriers need to be overcome for successful integration of novel immunotherapies: Toxicity and financial cost. The clinical use of the checkpoint inhibition is associated with a risk for serious, potentially fatal immune-related adverse events (irAEs). Following this paradigm, the ability to inhibit multiple targets simultaneously may be limited by the adverse event profile of such combinations. It is important to note that while it is unclear whether the same molecular mechanisms that drive tumor rejection are to blame for the induction of irAEs, both retrospective [353] and limited prospective data [354] show a correlation between irAEs and better outcomes. This correlation has not been adequately studied if it also concerns combinatorial immunotherapy, which is associated with a higher risk for severe irAEs according to the aforementioned CheckMate 067 trial [350].

On the other hand, the revolution of cancer immunotherapy has brought to the limelight the associated financial costs. Published data indicate that the combination of nivolumab and ipilimumab, despite its efficacy, is not a cost-effective option [355]. How quickly and widely the combination will be adopted in light of the positive results from randomized trials on malignancies that can be readily treated with other options [356,357], remains to be seen. It is reasonable to assume that future combinations with novel agents will not differ in that respect. In addition, the evaluation of novel combinations will likely be plagued by the same problems that have affected PD-1/PD-L1 inhibitors: Inconclusive predictive biomarkers lacking analytical validity and clinical validity/utility, variety of companion diagnostics using different antibodies and cut-offs, trials reporting different results from different antibodies in the same clinical setting and overabundance of available options with no hints on their differential efficacy [7]. It is therefore imperative that future phase 3 trials will be based on robust preclinical and early clinical data. 

## 8. Conclusions

A large number of co-stimulatory or co-inhibitory molecules regulating tumor evasion from immunosurveillance have been studied in BC (Table 5). As reviewed here, there are solid pre-clinical data on the function of these factors and emerging data on their regulation and their role in the clinical setting. These molecules likely represent future targets of immunotherapy provided that the promise shown in early data is translated into improved patient survival in randomized trials.

While it seems counterintuitive that the development of the next generation of immunotherapy agents precedes the optimization of the currently available ones, early recognition of the most promising agents can hasten their implementation in clinical practice. As we previously characterized the emergence of the PD-1/PD-L1 inhibition as the “end of the beginning” of cancer immunotherapy [7], the exciting advances that are described in this review could very well represent the “beginning of the end” of non-selective cytotoxic chemotherapy. 

## Figures and Tables

**Table 1:** Expression and prognostic/predictive value of immune-related markers predominantly expressed on T-cells.

Marker	BC Subtype	Number of Patients	Method	Positive/ Overexpressing Cases	Prognostic/Predictive Value	Comments	Reference
**LAG-3**	All	8	RT-PCR	LAG-3 expression: 8/8 (100%)	NA	LAG-3 overexpression in BC compared to adjacent healthy tissue	[23]
	All	148 pre-NACT 114 post-NACT	IHC	LAG-3 positivity: Pre-NACT: 33/148 (22.3%) Post-NACT: 38/114 (33.3%)	LAG-3 expression: pedictive for pCR in UA but not MA	Positive case cut-off: expression ≥ 5%	[28]
	TNBC	259 (training set) 104 (validation set)	IHC	LAG-3 positivity: 65/363 (18%)	LAG-3 positivity: trend to better RFS and OS in UA	Positive case cut-off: expression ≥ 5%	[25]
	All	330 (training set) 3992 (validation set)	IHC	LAG-3 positivity: 327/2921 (11%)	LAG-3 positivity: better BCSS and RFS in MA but not when considering CD8, PD-1 and PD-L1	Positive case cut-off: ≥ 1 TILs per TMA core 2921 evaluable in validation set	[26]
**TIM-3**	All	150	IHC	BC cases: TIM-3 + tumor cells 147/150 (98%) TIM-3+CD8+ T cells 135/150 (90%)Healthy controls: TIM-3+ epithelial cells 13/100 (13%) TIM-3+CD8+ T-cells 23/100 (23%)	NA		[29]
	All	20	FC	NA	NA	Peripheral blood: overexpression of TIM-3 in CD4+CXCR5+ICOS+ T cells compared to healthy controls TILs: overexpression of TIM-3 in CD4+CXCR5+ICOS+ T cells compared to peripheral blood of same patients	[30]
	All	8	RT-PCR	TIM-3 expression: 8/8 (100%)	NA	Overexpression in BC compared to healthy adjacent tissue	[23]
	All	3169	Gene expression dataset	No overexpression	NA	Use of gene expression dataset *Genevestigator v3*	[31]
	All	3992 (3148 evaluable)	IHC	TIM-3+ iTILs: 332/3148 (11%) TIM-3+ sTILs: 630/3148	TIM-3+ iTILs: better BCSS TIM-3+ sTILs: statistically not significant better BCSS	TIM-3 iTILs cut-off: expression ≥ 1 iTIL TIM-3 sTILs cut-off: expression ≥ 2 sTILs TIM-3+ iTILs correlated to basal-like subtype	[32]
**VISTA**	NA	NA	NA	NA	NA	NA	NA
**TIGIT**	All	3169	Gene expression dataset	TIGIT overexpression in 72%	NA	Use of gene expression dataset *Genevestigator v3*	[31]
	TNBC	47	Gene expression dataset	NA	TIGIT overexpression: better RFS and OS	Use of gene expression dataset from GEO datasets (GDS2250 and GSE3744)	[33]
	All	8	RT-PCR	TIGIT expression: 8/8 (100%)	NA	No overexpression of TIGIT in BC compared to adjacent healthy tissue	[23]
**GITR**	All	33	FC	NA	NA	PT Tregs: 80.5% expression of GITR Circulating Tregs: 28.9% expression of GITR	[34]
	All	39	FC	NA	NA	PT CD4+h T cells: higher GITR expression than healthy control CD4+ T cells	[35]
	All	3169	Gene expression dataset	GITR overexpression in 42%	NA	Use of gene expression dataset *Genevestigator v3*	[31]
	Not specified	3	FC	NA	NA		[36]
	Not specified	17	FC	NA	NA	More T regs expressing GITR in BC patients than healthy donors (*n* = 10)	[37]
**B7-H3**	All	221	IHC	B7-H3 high expression: Healthy controls: 14/85 (16.48%) BC: 178/221 (80.55%)	NA		[38]
	All	82	RT-PCR	B7-H3 overexpression: 32/82 (39%)	NA		[39]
	All	117	IHC	B7-H3 positivity: 106/117 (90.6%)	NA	Positive case cut-off: expression > 10%	[40]
	All	90	IHC	B7-H3 high: 83/90 (92%)	B7-H3 high: worse RFS but no association with OS		[41]
	All	74	IHC	B7-H3 IHC positivity: BC 42/74 (56.8%) healthy controls 32/74 (43.2%)	B7-H3 positivity: worse OS		[42]
	All	97	IHC	NA	NA	B7-H3 expression significantly higher in BC (*n*=97) compared to normal tissue (*n* = 53), benign, and precursor lesion (*n* = 182)	[43]
	All	208	IHC	B7-H3 positivity: BC: 154/208 (74%) Healthy controls: 3/7 (43%)	NA		[44]
	All	101	IHC	B7-H3 positivity: BC: 88/101 (88%) Healthy controls: 6/47 (12.8%)	NA		[45]
**ICOS**	All	120	IHC FC	NA	ICOS positivity: UA: worse PFS and OS MA: not significant	Positive case cut-off: expression ≥1.7 positive cells Tumoral Treg ICOS+: 69.9% BC circulating Treg ICOS+: 16.6% Healthy circulating Treg ICOS+: 21.3%	[46]
**4-1BB**	All	3169	Gene expression dataset	4-1BB overexpression in 42%	NA	Use of gene expression dataset *Genevestigator v3*	[31]
	All	286	Gene expression dataset	NA	4-1BB expression: better DFMS		[47]
	Not specified	4	IHC	4-1BB positivity: 2/4 (50%)	NA	Positive case cut-off: expression > 10%	[48]
**CD70**	All	204	IHC	CD70 positivity: 5/204 (2.45%)	NA		[49]
	All	139 (110/139 with metastasis) 233 (stage I – III)	IHC	CD70 expression: 81/139 (58.3%)	CD70 expression: worse lung MFS		[50]
	All	16 (pre and post-NACT)	RT-PCR	NA	CD70 overexpression after NACT: better PFS		[51]
**OX40 and OX40L**	All	107 9 DICS	IHC	Positivity in PT: OX40 91/107 (85%) OX40L 89/107 (83.2%) Positivity in DCIS: OX40 6/9 (66.7%) OX40L 7/9 (77.8%)	NA	Positive case cut-off: expression on > 10% tumor cells OX40 associated with advanced stage	[52]
	Not specified	19	IHC	OX40 positivity: 10/19 (52.6%)	NA	Positive case cut-off: expression on > 10% cells	[53]
	Not specified	Not specified	IHC FC	OX40+CD4+ TILs in 43% of the BC cases	NA	No OX40 expression on circulation CD4 T cells	[54]
	Not specified	45	IHC	OX40 positivity: 7/45 (15.55%)		OX40 expressed on TILs OX40 expression also found on positive LN	[55]
	Not specified	44	IHC	OX40 positivity: 7/18 (30%) of theCD4+ cases	NA		[56]
**BTLA**	All	3080	Gene-expression dataset	BTLA overexpressed in TNBC compared to non-TNBC	BTLA overexpression in TNBC: better OS and DFS	Use of gene expression profiles of breast invasive carcinoma from TCGA and METABRIC	[57]
	All	660	IHC FC	BTLA positivity: 15/660 (2.3%)	NA	Positive case cut-off: ≥ 1 BTLA+ TIL All BTLA+ TILs also expressed PD-1 According to FC, CD4 and CD8+TILs don’t express BTLA	[58]
**TLR9**	TNBC (Afro-American population)	51	IHC	TLR9 ”low” expression: 27/51 (52.9%) TLR9 ”high” expression: 22/51 (43.1%)	TLR9 high: no association with recurrence or BCSS	Variants of TLR9 gene associated with protection from breast cancer	[59]
	All and TNBC	84 of all subtypes 80 TNBC 350 of all subtypes	RT- PCRIHC	mRNA expression in cohort of 84 cases of all subtypes: overexpression in TNBC IHC expression in sub-group analyses of 38/84 cases of all subtypes: overexpression in 8/38 (21%) and 5/13 (38.5%) TNBC IHC expression in 80 TNBC cases: 32/80 (40%) mRNA expression in 350 cases of all subtypes: overexpression in 50/350 (14.3%) and 19/64 (29.7%) TNBC	High mRNA expression: trend to better MFS High protein expression in 80 TNBC: better MFS	TLR9 also expressed in pre-invasive lesions	[60]
	All	196	IHC	TLR9 high expression in TNBC: 51/99 (51.5%)	TLR9 high expression: All subtypes: no association found TNBC: high expression better BCSS		[61]
	All	12	RT-PCR	TLR9 expression: 12/12 (100%)	NA		[62]
	All	124	IHC	TLR9 positivity: 78/124 (63%)	TLR9 positivity: UA: worse PFS MA: not statistically significant	Positive case cut-off: expression > 10% cells Expression significantly higher in tumors with positive axillary LN metastasis, ER- and advanced stage	[63]
	All	74	IHC	TLR9 expression: By tumor cells: 73/74 (98.6%) By fibroblasts 42/74 (58%)	TLR9 positive expression by fibroblasts: better DMFS		[64]
	All	124 116 post-menopausal	RT-PCR IHC	TLR9 mRNA: overexpression in ER- TLR9 IHC expression in 116 post-menopausal: 103/116 (88.8%)		IHC expression higher in ER and PR-	[65]
	All	141	IHC	TLR9 positivity: 136/141 (98%)	TLR9 positivity: worse DMFS	Higher expression in ER- and high grade tumors	[66]
**A2aR**	NA	NA	NA	NA	NA	NA	NA
**CD73**	All	80	IHC	NA	CD73 expresion in ER+ cases: no prognostic value CD73 expression in ER- cases: worse OS	CD73 expression associated with EGFR expression	[67]
	All	136	IHC	CD73 positivity: 101/136 (74%)	CD73 positivity: UA: better DFS and OS MA: better DFS, trend to better OS		[68]
	All (Her2 status NA)	102	IHC	CD73 positivity: 9/102 (9%)	NA	Positive case cut-off: any expression by tumor cells	[69]
	Not specified	74	IHC	CD73 positivity: 60/74 (81%)	NA	Positive case cut-off: expression >5% cells	[70]
	TNBC	122	IF	NA	Tumor cells CD73 expression: UA: worse DFS and OS MA: worse DFS, trend to worse OS Stromal and immune CD73 expression: no prognostic value		[71]
	All	119	IHC	CD73 positivity: 100/119 (84%)	NA		[72]
	All	202	Gene expression dataset	NA	Gene-expression database of 1128 cases of all subtypes: worse DFS Gene-expression of 417 Her2+ cases: worse DFS Gene-expression of 784 ER+ and 211 TNBC cases: statistically NS trend to worse DFS METABRIC cohort of 1981 cases of all subtypes: worse DSS		[73]
	All and TNBC	6209 all subtypes 59 TNBC	Gene expression dataset	NA	6209 cases of all subtypes: worse OS for TNBC, no prognostic value for ER+ and Her2+ cases 59 TNBC: worse response to NACT		[74]
**CD39**	Not specified	33	FC	PT CD39+CD8+ TILs mean frequency: 18.5% +/− 4.3% Circulating CD8+ T cells: no CD39 expression	NA		[75]
	All (Her2 NA)	11	FC	NA	NA	CD39+CD4+ TILs 28.7+/−5.8% vs 8.2+/−5.9% in normal adjacent tissue CD39+CD8+ TILs 9+/−3.5% vs 0.4+/−0.3% in normal adjacent tissue	[76]
	All	50	FC IF RT-PCR	NA	NA	CD39 +Th17 TILs 93.6% CD39 + TILs Tregs 50.9% CD39 overexpressed among IL-17^Hi^ tumors	[77]
	All	3169	Gene expression dataset	No CD39 overexpression	NA	Use of gene expression dataset *Genevestigator v3*	[31]
	Not specified	10	Gene expression dataset	CD39 overexpressed in BC compared to healthy tissue	NA	Micro-array dataset from *Turashvili et al.* (BMC Cancer. 2007 Mar 27;7:55.)	[78]

**Abbreviations:** LAG-3, lymphocyte-activation gene 3; TIM-3, T-cell immunoglobulin and mucin-domain containing-3; VISTA, V-domain Ig suppressor of T cell activation; TIGIT, T-cell immunoreceptor with Ig and ITIM domains; GITR, glucocorticoid-induced TNFR-related protein; B7-H3, B7 homolog 3; ICOS, Inducible T-cell costimulator; 4-1BB; CD70, cluster of differentiation 70; BTLA, B- and T-lymphocyte attenuator; TLR9, Toll-like receptor 9; A2aR, A2A adenosine receptor; CD73, cluster of differentiation 73; CD39, cluster of differentiation 39; BC, breast cancer; TNBC, triple-negative breast cancer; Her2, human epidermal growth factor receptor 2; NACT, neo-adjuvant chemotherapy; RT-PCR, reverse transcription polymerase chain reaction; IHC, immunohistochemistry; FC, flow-cytometry; IF, immunofluorescence; mRNA, messenger RNA; TILs, tumor-infiltrating lymphocytes; NA, not assessed; UA, univariate analysis; MA, multivariate analysis; pCR, pathological complete response; RFS, relapse-free survival; OS, overall survival; BCSS, breast cancer specific survival; PD-1, Programmed cell death 1; PD-L1, Programmed death-ligand 1; DFMS, distant-metastasis free survival; MFS, metastasis-free survival; PFS, progression-free survival; DFS, disease-free survival; ER, estrogen receptor; PR, progesterone receptor; NS, non significant; DSS, disease-specific survival; TMA, tissue microarray; Tregs, regulatory T cells; LN, lymph-node; TCGA, the cancer genome atlas; EGFR, epidermal growth factor receptor.

**Table 2:** Ongoing clinical trials potentially including breast cancer patients for targeting immune-related markers predominantly expressed on T-cells.

Target	Drug	Other Agent(s)	Phase	Disease	Line	NCT Identifier	Trial Status
LAG-3	IMP 321 (Eftilagimod)	+ Paclitaxel	I/II	Advanced BC	1st line	NCT00349934	Completed, published results [27]
		+ Paclitaxel	Iib	Hormone positive advanced BC	1st line	NCT02614833	Recruiting, safety results published [79]
		+ Paclitaxel	I	Advanced BC (chinese population)	1st line	NCT03600090	Not yet recruiting
		+ standard therapy	I	Advanced solid tumors	Any line	NCT03252938	Recruiting
	MK-4280	+/− Pembrolizumab (anti-PD1)	I	Advanced solid tumors	No standard therapy available	NCT02720068	Recruiting
	BMS-986016 (Relatlimab)	+/− Nivolumab (anti-PD1)	I	Advanced solid tumors	No standard therapy available	NCT02966548	Recruiting
		+ Nivolumab (anti-PD1) and BMS-986205 (IDO1 inhibitor) Or + Nivolumab (anti-PD1) and Ipilimumab (anti-CTLA4)	I/II	Advanced solid tumors	Any line	NCT03459222	Recruiting
	REGN3767	+/− REGN2810 (anti-PD1)	I	Advanced solid tumors	No standard therapy available	NCT03005782	Recruiting
	LAG525 (IMP701)	+/− PDR001 (anti-PD1)	I/II	Advanced solid tumors including TNBC	≥ 1 line	NCT02460224	Active, not recruiting Preliminary results published [80]
		+/− PDR001 (anti-PD1) +/− Carboplatin	II	Advanced TNBC	1^st^ or 2^nd^ line	NCT03499899	Suspended
		+ PDR001 (anti-PD1) + NIR178 (A2aR antagonist) or Capmantinib (C-MET inhibitor) or MCS110 (anti-M-CSF) or Canakinumab (anti-IL1)	I/Ib	TNBC	≤ 2 lines	NCT03742349	Recruiting
	TSR-033	+ anti-PD1	I	Advanced solid tumors	No standard therapy available	NCT03250832	Recruiting
	INCAGN02385	No	I	Advanced solid tumors including TNBC	No standard therapy available	NCT03538028	Not yet recruiting
	Sym022	No	I	Advanced solid tumors	No standard therapy available	NCT03489369	Recruiting
		+ Sym021 (anti-PD1) or Sym023 (anti-TIM3)	I	Advanced solid tumors	No standard therapy available	NCT03311412	Recruiting
	MGD013 (Anti- LAG3 + Anti-PD1)	No	I	Advanced solid tumors	No standard therapy available	NCT03219268	Recruiting
	FS118 (Anti-LAG3 + Anti-PDL1)	No	I	Advanced solid tumors that progressed on anti-PD1/PDL-1 therapy	≥ 1 line	NCT03440437	Recruiting
	XmAb®22841 (Anti- LAG3 + Anti-CTLA4)	No	I	Advanced solid tumors including TNBC	No standard therapy available	NCT03849469	Not yet recruiting
TIM-3	MBG453	+/− PDR001 (anti-PD1)	I-Ib/II	Advanced solid tumors (phase I)	No standard therapy available	NCT02608268	Recruiting
	TSR-022	No	I	Advanced solid tumors	No standard therapy available	NCT02817633	Recruiting
		+ Carboplatin + Nab-paclitaxel + TSR-042 (anti-PD1)	I	Advanced solid tumors	≤ 1 line (part B) ≤ 4 lines (part A)	NCT03307785	Recruiting
	LY3321367	+/− LY3300054 (anti-PDL1)	Ia/Ib	Advanced solid tumors	No standard therapy available	NCT03099109	Recruiting
	INCAGN02390	No	I	Advanced solid tumors including TNBC	No standard therapy availaible	NCT03652077	Recruiting
	Sym023	No	I	Advanced solid tumors	No standard therapy availaible	NCT03489343	Recruiting
		+ Sym021 (anti-PD1) or Sym022 (anti-LAG3)	I	Advanced solid tumors	No standard therapy available	NCT03311412	Recruiting
	LY3321367	+/− LY3300054 (anti-PDL1)	I	Advanced solid tumors	Any line	NCT03099109	Recruiting
	BGB-A425	+/− Tislelizumab (anti-PD1) for phase II	I/II	Advanced solid tumors	No standard therapy available	NCT03744468	Recruiting
	LY3415244 (Anti-TIM3 + Anti-PDL1)	No	Ia/Ib	Advanced solid tumors	Any line (phase Ia) ≥ 1 line with anti-PD1 or anti-PDL1 therapy (phase Ib)	NCT03752177	Recruiting
	MBG453	+ PDR001 (anti-PD1)	I/II	Advanced solid tumors	No standard therapy available and no prior anti-PD1/PDL1 therapy	NCT02608268	Recruiting
VISTA	CA-170	No	I	Advanced solid tumors including TNBC	No standard therapy availaible	NCT02812875	Recruiting
TIGIT	AB154	+/− AB122 (anti-PD1)	I	Advanced solid tumors	No standard therapy availaible	NCT03628677	Recruiting
	OMP-313M32 (Etigilimab)	+/− Nivolumab (anti-PD1)	Ia/Ib	Advanced solid tumors	No standard therapy availaible	NCT03119428	Active, not recruiting
	BMS-986207	+/− Nivolumab (anti-PD1)	I/II	Advanced solid tumors	No standard therapy availaible	NCT02913313	Recruiting
GITR	MK-4166	+/− Pembrolizumab (anti-PD1)	I	Advanced solid tumors	No standard therapy availaible	NCT02132754	Active, not recruiting
	INCAGN01876	+/− Epacadostat (IDO1 inhibitor) +/− Pembrolizumab (anti-PD1)	I/II	Advanced solid tumors (phase I)	No standard therapy availaible	NCT03277352	Active, not recruiting
		+/− Nivolumab (anti-PD1) +/− Ipilimumab (anti-CTLA4)	I/II	Advanced solid tumors (phase I)	No standard therapy availaible	NCT03126110	Recruiting
		No	I/II	Advanced solid tumors (phase I)	No standard therapy availaible	NCT02697591	Recuiting
	TRX518	+/− Gemcitabine +/− Pembrolizumab (anti-PD1) +/− Nivolumab (anti-PD1)	I	Advanced solid tumors (monotherapy and association with Gemcitabine)	No standard therapy availaible or indication for Gemcitabine	NCT02628574	Recruiting
		No	I	Advanced solid tumors	No standard therapy availaible	NCT01239134	Recruiting, safety results published [81]
		+ Cyclophosphamide and/or Avelumab (anti-PDL1)	I/II	Advanced solid tumors including TNBC and hormone receptor positive refractory BC	TNBC: 2nd or 3rd line Hormone receptor positive BC: ≥ 1 line with aromatase inhibitor	NCT03861403	Not yet recruiting
	BMS-986156	+/− Nivolumab (anti-PD1)	I/Iia	Advanced solid tumors	No standard therapy availaible	NCT02598960	Active, not recruiting preliminary results [82]
		+/− Nivolumab (anti-PD1)	I	Advanced solid tumors	≥ 2 lines	NCT03335540	Recruiting
	GWN323	+/− PDR001 (anti-PD1)	I/Ib	Advanced solid tumors	Not specified	NCT02740270	Active, not recruiting
	MEDI1873	No	I	Advanced solid tumors	Not specified	NCT02583165	Completed, no published results
	OMP-336B11	No	Ia	Advanced solid tumors	No standard therapy availaible	NCT03295942	Active, not recruiting
B7-H3	MGA271 (Enoblituzumab)	+/− Pembrolizumab (anti-PD1)	I	Advanced solid tumors including TNBC	No standard therapy available	NCT02475213	Active, not recruiting
		+ Ipilimumab (anti-CTLA4)	I	Advanced solid tumors including TNBC	No standard therapy available	NCT02381314	Active, not recruiting
	MGD009 (Orlotamab)	No	I	Advanced solid tumors including TNBC	≥ 1 prior line	NCT02628535	Recruiting
		MGA012 (anti-PD1)	I	Advanced solid tumors expressing B7-H3	No standard therapy available	NCT03406949	Recruiting
	MGC018	+/− MGA012 (anti-PD1)	I/II	Advanced solid tumors	No standard therapy available	NCT03729596	Recruiting
ICOS	JTX-2011	+/− Nivolumab (anti-PD1) +/− Ipilimumab (anti-CTLA4) +/− Pembrolizumab (anti-PD1)	I/II	Advanced solid tumors	No standard therapy availaible	NCT02904226	Recruiting, safety results published [83]
	BMS-986226	+/− Nivolumab (anti-PD1) or Ipilimumab (anti-CTLA4)	I/II	Advanced solid tumors	≥ 1 prior line	NCT03251924	Recruiting
4-1BB	PF-05082566 (Utolimumab)	+ Trastuzumab – Vinorelbine – Avelumab (anti-PDL1) + Trastuzumab – Avelumab (anti-PDL1)	II	Advanced Her2+ BC	≥ 1 prior line with progression under Trastuzumab - Pertuzumab	NCT03414658	Recruiting
		Cohort 1: + Trastuzumab – Emtansine Cohort 2: + Trastuzumab	IB	Advanced Her2+ BC	Cohort 1: ≥ 1 prior line with taxane and trastuzumab Cohort 2: ≥ 2 prior lines	NCT03364348	Recruiting
		+ Avelumab (anti-PDL1)	IB/II	Advanced solid tumors including TNBC	Any line	NCT02554812	Recruiting
		Arm A: + Avelumab (Anti-PD-L1) Arm C: + Avemulmab (anti-PD-L1) and PF-04518600 (anti-OX40)	I/II	Advanced solid tumors	No strandard therapy available	NCT03217747	Recruiting
	BMS-663513 (Urelumab)	+/− Nivolumab (anti-PD1)	I/II	Advanced solid tumors	Any line	NCT02253992	Active, not recruiting
		+ SBRT – Nivolumab (anti-PD1)	I	Advanced solid tumors	Any line	NCT03431948	Recruiting
		No	I	Advanced solid tumors	No strandard therapy available	NCT01471210	Completed, preliminary safety results published [84]
		+ Nivolumab (anti-PD1)	I	Advanced solid tumors	No strandard therapy available	NCT02534506	Active, not recruiting
		+ Nivolumab (anti-PD1)	I/II	Advanced solid tumors	No strandard therapy available	NCT03792724	Not yet recruiting
	PRS-343	+ Atezolizumab (anti-PDL1)	IB	Advanced solid tumors including Her2+ BC	≥ 2^nd^ line	NCT03650348	Recruiting
		No	I	Advanced solid tumors including Her2+ BC	No strandard therapy available	NCT03330561	Recruiting
	ADG106	No	I	Advanced solid tumors	No strandard therapy available	NCT03802955	Recruiting
		No	I	Advanced solid tumors	No strandard therapy available	NCT03707093	Recruiting
CD27/CD70	Anti-hCD70 CAR PBL	+ Aldeskeukin (IL-2)	I/II	Advanced solid tumors expressing CD70	≥ 2^nd^ line	NCT02830724	Recruiting
	ARGX-110 (Cusatuzumab)	No	I/II	Advanced solid tumors expressing CD70	No standard therapy available	NCT01813539	Active, not recruiting Safety results published [85]
	CDX-1127 (Varlilumab)	+ ONT-10 (Immunovaccine)	IB	Advanced BC	≥ 2^nd^ line	NCT02270372	Completed, no published results
OX40/OX40L	MOXR0916 (Vonlerolizumab)	No	I	Advanced solid tumors	No standard therapy available	NCT02219724	Active, not recruiting
		+ Atezolizumab (anti-PDL1)	IB	Advanced solid tumors	No standard therapy available	NCT02410512	Active, not recruiting Preliminary safety results published [86]
	PF-04518600	+ Avelumab (anti-PDL1) Or + Utolilumab (Anti-4-1BB) and Avelumab (anti-PDL1) +/− Radiation	I/II	Advanced solid tumors	No standard therapy available	NCT03217747	Recruiting
	MEDI6383	+/− MEDI4736 (anti-PDL1)	I	Advanced solid tumors	No standard therapy available ≤ 5 prior lines	NCT02221960	Completed, no published results
	MEDI0562	+/− MEDI4736 (anti-PDL1) Or +/− Tremelilumab (anti-CTLA4)	I	Advanced solid tumors	No standard therapy available ≤ 3 prior lines	NCT02705482	Active, not recruiting
	INCAGN01949	No	I/II	Advanced solid tumors	No standard therapy available	NCT02923349	Active, not recruiting
		+/− Nivolumab (anti-PD1) +/− Ipilimumab (anti-CTLA4)	I/II	Advanced solid tumors (phase I)	No standard therapy available	NCT03241173	Active, not recruiting
	GSK3174998	+/− Pembrolizumab (anti-PD1)	I	Advanced solid tumors	No standard therapy available ≤ 5 prior lines	NCT02528357	Recruiting
		+ GSK1795091 (TLR4 agonist)	I	Advanced solid tumors including BC but not TNBC	No standard therapy available	NCT03447314	Recruiting
	MEDI6469	+ SBRT to liver or lung metastases	I/II	Advanced BC	≥ 1 prior line	NCT01862900	Completed, no published results
	mRNA 2416	No	I	Advanced solid tumors	No standard therapy available	NCT03323398	Recruiting
	BMS-986178	+ intra-tumoral SD-101 (TLR9 agonist)	I	Advanced solid tumors	≥ 1 prior line	NCT03831295	Recruiting
		+/− Nivolumab (anti-PD1) and/or Ipilimumab (anti-CTLA4)	I/IIa	Advanced solid tumors	≥ 1 prior line	NCT02737475	Recruiting
BTLA	NA	NA	NA	NA	NA	NA	NA
TLR9	IMO-2125 (Tilsotolomid) Intra-tumoral	No	Ib	Advanced solid tumors	Any line (previously treated with anti-PDL1 therapy if indicated)	NCT03052205	Active, not recruiting Preliminary safety results published [87]
	Agatolimod (CPG 7909; PF-3512676)	+ Trastuzumab	I/II	Advanced Her2+ BC	≤ 3 lines	NCT00043394	Completed, no published results
		+ Trastuzumab	I/II	Advanced Her2+ BC	Not specified	NCT00031278	Completed, no published results
		+ Montanide® ISA-51 (immune modulator) + NY-ESO-l protein (therapeutic vaccine)	I	Localised solid tumors	Neo-adjuvant or adjuvant chemotherapy authorised	NCT00299728	Completed, no published results
		+ Montanide ISA 720 (immune modulator) + Cyclophosphamide + NY-ESO-1-derived Peptides or Protein (therapeutic vaccine)	I	Advanced solid tumors expressing NY-ESO-1	≥ 2^nd^ line	NCT00819806	Completed, no results published
	MGN1703	+ Ipilimumab (anti-CTLA4)	I	Advanced solid tumors	No standard therapy available	NCT02668770	Recruiting
	SD-101	+ BMS 986178 (anti-OX40)	I	Advanced solid tumors	≥ 1 prior line	NCT03831295	Recruiting
		+ Pembrolizumab (anti-PD1)	II	Stage II or III BC	No prior treatment	NCT01042379	Recruiting
**Adenosine pathway**							
A2aR	NIR178	+/− NZV930 (anti-CD73) +/− PDR001 (anti-PD1)	I/IB	Advanced solid tumors including TNBC	No standard therapy available	NCT03549000	Recruiting
		+ PDR001 (anti-PD1) and LAG525 (anti-LAG3)	I	Advanced TNBC	≤ 2 prior lines	NCT03742349	Recruiting
	AZD4635	+/− Durvalumab (Anti-PDL1)	I	Advanced solid tumors	No standard therapy available	NCT02740985	Recruiting
	AB928	+ AB122 (anti-PD1)	I	Advanced solid tumors	No standard therapy available	NCT03629756	Recruiting
		+/− Pegylated liposomal doxorubicin	I/Ib	Advanced TNBC	No standard therapy available	NCT03719326	Recruiting
	CPI-444	+/− Atezolizumab (anti-PDL1)	I	Advanced solid tumors including TNBC	≥ 1 and ≤ 5 prior lines	NCT02655822	Recruiting
		+/− CPI-006 (anti-CD73)	I/IB	Advanced solid tumors including TNBC	≥ 1 and ≤ 5 prior lines	NCT03454451	Recruiting
CD73	SRF373 (NZV930)	+/− PDR001 (anti-PD1) +/− NIR178 (A2aR antagonist)	I/IB	Advanced solid tumors including TNBC	No standard therapy available	NCT03549000	Recruiting
	CPI-006	+/− CPI-444 (A2aR antagonist) +/− Pembrolizumab (anti-PD1)	I/IB	Advanced solid tumors including TNBC	≥ 1 and ≤ 5 prior lines	NCT03454451	Recruiting
	BMS-986179	+/− Nivolumab (anti-PD1) +/− rHuPH20 (Recombinant human hyaluronidase)	I/IIA	Advanced solid tumors	Any line	NCT02754141	Recruiting, preliminary results published [88]
	MEDI9447 (Oleclumab)	+/− MEDI4736 (anti-PDL1)	I	Advanced solid tumors	Any line	NCT02503774	Recruiting
		+ Paclitaxel – Carboplatin – Durvalumab (anti-PDL1)	I/II	Advanced TNBC	1st line	NCT03616886	Recruiting
		No	I	Advanced solid tumors (Japanese population)	No standard therapy available	NCT03736473	Active, not recruiting
		+ NACT + pre-operative surgery + Durvalumab (anti-PDL1)	II	Luminal B BC (neo-adjuvant setting)	Neo-adjuvant setting	NCT03875573	Not yet recruiting
		+ Paclitaxel + Durvalumab (anti-PDL1)	I/II	Advanced TNBC	1st line	NCT03742102	Recruiting
CD39	NA	NA	NA	NA	NA	NA	NA

**Abbreviations:** LAG-3, lymphocyte-activation gene 3; TIM-3, T-cell immunoglobulin and mucin-domain containing-3; VISTA, V-domain Ig suppressor of T cell activation; TIGIT, T-cell immunoreceptor with Ig and ITIM domains; GITR, glucocorticoid-induced TNFR-related protein; B7-H3, B7 homolog 3; ICOS, Inducible T-cell costimulator; 4-1BB; CD27, cluster of differentiation 27; CD70, cluster of differentiation 70; BTLA, B- and T-lymphocyte attenuator; TLR9, Toll-like receptor 9; A2aR, A2A adenosine receptor; CD73, cluster of differentiation 73; CD39, cluster of differentiation 39; PD1, Programmed cell death 1; IDO1, Indoleamine 2, 3-dioxygenase 1; CTLA4, Cytotoxic T-Lymphocyte Associated Protein 4; PDL1, Programmed death-ligand 1, IL-2, Interleukine-2; SBRT, Stereotactic Body Radiation Therapy; NACT, neo-adjuvant chemotherapy; BC, breast cancer; TNBC, triple-negative breast cancer; Her2, human epidermal growth factor receptor 2.

**Table 3:** Expression and prognostic/predictive value of immune-related markers predominantly expressed by macrophages, NK and dendritic-cells in breast cancer (BC) patients.

Marker	BC Subtype	Number of Patients	Method	Positive/Overexpressing Cases	Prognostic/Predictive value	Comments	Reference
**Macrophage-related**							
CSF-1/CSF-1R	All	581 (301 node-negative, 280 node-positive)	IHC	Positive cases: node-negative 114/301 (38.9%) node-positive 189/280 (67.5%)	Positivity in node negative: worse OS (not in node positive patients)		[264]
	All	196	IHC in situ RNA detection	74% CSF-1+ and 58% CSF-1R+ tumors	CSF-1+ tumor cells: poor survival	CSF-1+ tumor cells: more frequent metastases	[263]
	All	572	ELISA (circulating CSF1 levels)	NA	logCSF1: worse BCCS high CSF-1: worse outcome in post-menopausal patients	Cut-off: median serum CSF-1 expression	[262]
	All	68	IHC	NA	High CSF-1: worse DSS	High CSF-1R: marginally correlated to worse DSS	[261]
CCL2/CCR2	All	137	IHC	CCL2+ tumor cells: 30.7% in PTs vs 39.4% in paired recurrences CCL2+ stromal cells: 18.2% in PTs vs 22.6% in paired recurrences	No correlation	Significantly higher CCL2 expression in tumor cells of recurrences (especially the early ones) compared to PTs	[267]
	All	427	IHC	NA	Stromal but not epithelial CCL2 expression: worse RFS in basal-like subtype	Stromal CCL2 remained an independent factor of worse prognosis in basal-like subtype	[268]
	All	63	IHC	NA	CCR2 expression in tumor cells: worse DFS, MFS and OS	CCR2 expression in tumor cells and CCL2 expression in stromal cells associated with higher risk of metastasis. CCR2 expression in tumor cells remained an independent factor of worse MFS	[269]
	All	151 (135 evaluable)	IHC	CCL2 high: 65/135 (48.1%) CCL2 low: 70/135 (51.9%)	CCL2 high: worse RFS	High combined CCL2/VEGF expression was independently associated with worse RFS	[270]
	All	3554 (TCGA and kmplot.com)	RNA-seq	NA	High mRNA CCL2 expression: better RFS in basal-like, HER2-enriched and luminal-B subtypes (median cutoff of mRNA expression)	No significant association between RFS and expression of CCL2 mRNA in the whole cohort and in luminal-A subtype	[271]
CD40	All	181	IHC	Cytoplasmic tumor cell expression: 53% Membrane tumor cell expression: 7.7% Nuclear tumor cell expression: 81%	CD40 cytoplasmic positivity: better OS	Positive association of CD40 cytoplasmic expression in HR+ breast tumors	[272]
**NK cell-related**							
CD94/NKG2A	All	28 (TDLN)	Flow cytometry	NA	NA	High expression of NKG2A in NK cells of tumor-draining lymph nodes described NKG2A+ NK cells correlated to locally advanced disease	[273]
NKG2D ligands (MICBAB, ULBP1-5)	All	677	IHC	Tumor cell expression: MIC-AB: 50% ULBP-1: 90% ULBP-2: 99% ULBP-3: 100% ULBP-4: 26% ULBP-5: 90%	High MIC-AB and ULBP-2 expression better RFS	Combined low expression of MIC-AB and ULBP-2 correlated to worse RFS	[274]
**Dendritic cell-related**							
IDO	All (Pakistani population)	100	IHC	100% positive 24/100 low IDO (24%) 27/100 medium IDO (27%) 49/100 high IDO (49%)	Medium and high IDO: worse OS	IDO expression correlated to TNBC	[275]
	All	203	IHC	100% positive 108/203 low IDO (53.2%) 95/203 intermediate and high IDO (46.8%)	General population: no difference in OS ER+ IDO intermediate/high: better OS Node-positive IDO intermediate/high: better DSS	IDO expression correlated to ER+	[276]
	All	26 primary tumor + TDLN 10 benign lesions	IHC	IDO positivity: PT: 12/26 (46.15%) TDLN: 19/26 (73.08%) Benign lesions: 1/10 (10%)	IDO expression: statistically not significant worse OS and TTP	IDO expression correlated to advanced stages, lymph-node metastasis and Treg infiltration No expression in healthy adjacent tissue	[277]
	All	155	IHC	Stromal positivity (>5%): 49/155 (31%) Epithelial positivity (>10%) 24/155 (15%)	IDO positivity: better OS	IDO positivity correlated to absence of lymph-node metastasis, ER- and TNBC	[278]
	All	242 primary tumor 20 TDLN 19 metastasis	IHC	IDO positivity: PT: 34/242 (14%) TDLN: 1/20 (5%) Metastasis: 0/19 (0%)	NA	IDO positivity correlated to high grade and TNBC Co-expression of IDO in 70% of PDL-1+ cases	[279]
	All	65	IHC	IDO positivity: 42/65 (64.6%)	IDO expression: worse OS and PFS in UA but not MA	IDO expression correlated to high grade, lymph-node metastastasis	[280]
	All	54	IHC	IDO positivity: 27/54 (68.5%)	IDO expression: worse response to NACT and statistically not significant worse PFS and OS	IDO expression correlated to advanced stages, lymph-node metastasis	[281]
	All	129 PT 10 normal LN 17 metastatic LN	IHC	IDO expression: PT: NA Normal lymph-nodes 80% Metastatic lymph nodes 88.2%	NA	IDO expression correlated to lymph-node metastasis, ER-, TNBC and PD-1 expression	[282]
	All	54 PT 11 healthy controls	qRT-PCR	NA	NA	IDO expression reduced in tumor compared to healthy tissue IDO expression in tumor correlated to advanced stage	[283]
	All	46	IHC	IDO high: 26/46 (56.5%)	IDO high: worse response to NACT and worse PFS and OS	IDO high correlated to advanced stage and lymph-node metastasis	[284]
	HR+	362	IHC	IDO expression 276/362 (76.2%)	IDO expression: worse OS	IDO expression not correlated to clinico-pathological characteristics IDO expression negatively correlated to B-cell infiltration	[285]
	All	202	IHC	NA	IDO high (expression by CAFs): worse DSS and MFS		[286]
	All	91 PT 21 benign lesions 10 healthy controls	IHC	IDO expression: PT: 55/91 (60%) Benign lesions 9/21 (43%) Healthy controls 2/10 (20%)	NA	IDO expression correlated to advanced stage	[287]
	All	85	IHC	NA	NA	IDO expression correlated to Treg infiltration and lymph-node metastasis	[288]
	All	5	IHC	IDO expression 5/5 (100%)	NA		[289]

**Abbreviations:** CSF-1R, colony-stimulating factor 1 receptor; CSF-1, colony-stimulating factor 1; CCL2, C-C Motif Chemokine Ligand 2; CCR2, C-C Motif Chemokine Receptor 2; IDO, Indoleamine 2,3-dioxygenase; NK-cells, natural-killer cells; CD40, cluster of differentiation 40; CD94, cluster of differentiation 94; NKG2A, NK group member 2A; NKG2D, NK group member 2D; VEGF, vascular endothelial growth factor; IHC, immunohistochemistry; qRT-PCR, quantitative real-time polymerase chain reaction; T-reg, T-regulatory cells; OS, overall survival; PFS, progression-free survival; MFS, metastasis-free survival; RFS, relapse-free survival; TTP, time-to-progression; DSS, disease-specific survival; BCCS, breast cancer-specific survival; PT, primary tumor; NACT, neoadjuvant chemotherapy; PD-1, programmed death 1; TNBC, triple-negative breast cancer; ER, estrogen receptor; HR, hormone receptor; CAFs, cancer-associated fibroblasts; MICBA/B, MHC class I chain-related protein A and B; ULBP1-5, UL binding protein 1-5; LN, lymph node; TDLN, tumor-draining lymph node; NA, not available.

**Table 4:** Ongoing clinical trials potentially including breast cancer patients for targeting of immune-related markers predominantly expressed on macrophages, NK and dendritic cells.

Target	Drug	Other Agent(s)	Phase	Disease	Line	NCT Identifier	Trial Status
**TAM-stimulatory markers**							
***CSF-1/CSF-1R***							
CSF-1R/CSF-1 inhibitors	PLX 3397 (Pexidartinib)	+ Eribulin	Ib/II	Metastatic breast cancer	≥ 1 prior line	NCT01596751	Active, not recruiting
		No	I	Advanced solid tumors	No standard therapy available	NCT01004861	Active, not recruiting
		+/− Paclitaxel	Ib	Advanced solid tumors	Not specified	NCT01525602	Completed, no published results
	ARRY-382	+/− Pembrolizumab (anti-PD1)	Ib/II	Advanced solid tumors including TNBC (phase Ib)	No standard therapy available	NCT02880371	Recruiting
		No	I	Advanced or metastatic solid tumors	No standard therapy available	NCT01316822	Completed, no published results
	BLZ945	+/− PDR001 (anti-PD1)	I	Advanced solid tumors including TNBC	Not specified	NCT02829723	Recruiting
Anti CSF-1R antibodies	LY3022855 (IMC-CS4)	No	I	Advanced BC	≥ 1 prior line	NCT02265536	Completed, no published results
		+ Durvalumab (anti-PDL1) or Tremelimumab (anti-CTLA4)	I	Advanced solid tumors	Not specified	NCT02718911	Completed, no published results
		No	I	Advanced solid tumors	No standard therapy available	NCT01346358	Completed, safety results published [290]
	RO5509554 (Emactuzumab)	+ Atezolizumab (anti-PDL1)	I	Advanced solid tumors including TNBC	Not specified	NCT02323191	Recruiting
		+/− Paclitaxel	I	Advanced solid tumors	No standard therapy available	NCT01494688	Completed, preliminary safety and activity results published [291]
		+ RO7009789 (CD40 agonist)	Ib	Advanced solid tumors including TNBC	No standard therapy available	NCT02760797	Completed, no published results
	AMG820	No	I	Advanced solid tumors	Not specified	NCT01444404	Completed, no published results
	SNDX-6352	Phase Ia: SNDX-6352 monotherapy Phase Ib: + Durvalumab (anti-PDL1)	I	Advanced solid tumors	≥ 1 prior line and no standard therapy available	NCT03238027	Recruiting
	Cabiralizumab (BMS-986227, FPA008)	+/− Nivolumab (anti-PD1)	I	Advanced malignancies	No standard therapy available	NCT03158272	Recruiting
		+ Nivolumab (anti-PD1) and SBRT	I	Advanced malignancies	No standard therapy available	NCT03431948	Recruiting
	PD 0360324 (M-CSF mAb)	+ Avelumab (anti-PDL1)	Ib/II	Advanced solid tumors including TNBC	No standard therapy available	NCT02554812	Recruiting
**CCL2/CCR2**							
CCR2 antagonist	PF-04136309	+ Avelumab (anti-PDL1) +Utomilumab (anti-4-1BB)	Ib/II	Advanced solid tumors including TNBC	No standard therapy available	NCT02554812	Recruiting
**CD47 – SIRPα**							
Anti-CD47 antibodies	Hu5F9-G4	+ Cetuximab (anti-EGFR)	Ib/II	Advanced solid tumors including BC (phase Ib)	≥ 1 prior line	NCT02953782	Recruiting
		+Avelumab (anti-PDL1)	Ib	Advanced solid tumors	Not specified	NCT03558139	Recruiting
	CC-90002	No	I	Advanced solid tumors	No standard therapy available	NCT02367196	Recruiting
	IBI188	No	Ia	Advanced solid tumors	No standard therapy available	NCT03763149	Recruiting
		No	I	Advanced solid tumors	No standard therapy available	NCT03717103	Recruiting
	AO-176	No	I	Advanced solid tumors	No standard therapy available	NCT03834948	Recruiting
	SRF231	No	I/Ib	Advanced solid tumors	No standard therapy available	NCT03512340	Recruiting
SIRPα-IgG1-Fc	TTI-621 (intra-tumoral injection)	+/− PD1/PDL1 Inhibitor	I	Advanced solid tumors with percutaneously accessible lesions	No standard therapy available	NCT02890368	Recruiting
	ALX148	+/− Trastuzumab or Pembrolizumab (anti-PD1) or Rituximab (anti-CD20)	I	Advanced solid tumors	No standard therapy available	NCT03013218	Recruiting, preliminary safety results published [292]
**TAM-inhibitory markers**							
CD40 (agonists)	CP-870,893	No	I	Advanced solid tumors	No standard therapy available	NCT02225002	Completed, no published results
		No	I	Advanced solid tumors	Patients who had clinical benefit following a single infusion of CP-870, 893	NCT02157831	Completed
	RO7009789 Selicrelumab	+ Atezolizumab (anti PDL1)	Ib	Advanced solid tumors	No standard therapy available	NCT02304393	Recruiting
		+ Emactuzumab (anti-CSF-1R)	I	Advanced solid tumors including TNBC	No standard therapy available	NCT02760797	Completed, no published results
		+ Vanucizumab (anti-VEGF-A and angiopoietin-2)	I	Metastatic solid tumors	Not specified	NCT02665416	Recruiting
	ADC-1013 (intra-tumoral or intra-venous injection)	No	I	Advanced solid tumors	Not specified	NCT02379741	Completed, no published results
	JNJ-64457107	No	I	Advanced solid tumors	Not specified	NCT0282909	Recruiting
TLR7 (agonists)	Imiquimod	+ Cyclophosphamide and Radiotherapy	I/II	Advanced BC with skin metastases	Any line	NCT01421017	Completed, no published results
**NK cell-inhibitory markers**							
CD94/ NKG2A	IPH2201	+ Durvalumab (anti-PDL1)	I/II	Advanced solid tumors	Any line	NCT02671435	Recruiting
KIR family	Lirilumab (anti-KIR2DL1,2,3 antibody)	+Nivolumab (anti-PD1) Or + Nivolumab (anti-PD1) and Ipilimumab (anti-CTLA4)	I	Advanced and/or metastatic solid tumors	Not specified	NCT03203876	Active, not recruiting
		+Nivolumab (anti-PD1)	I/II	Advanced solid tumors	≥ 1 and ≤ 5 prior lines	NCT01714739	Active, not recruiting
**IDO**							
Small-molecule inhibitor of IDO-1	Epacadostat (INCB024360)	+ INCB01158 (arginase inhibitor) +/− Pembrolizumab (anti-PD1)	I/II	Advanced solid tumors	No standard therapy available	NCT03361228	Active, not recruiting
		+ Pembrolizumab (anti-PD1)	I/II	Advanced or metastatic solid tumors including TNBC (phase I)	≥ 1 prior line	NCT02178722	Active, not recruiting Preliminary safety and efficacy results published [293]
		+ Sirolimus (mTOR inhinitor)	I	Advanced solid tumors	≥ 1 prior line and no standard therapy available	NCT03217669	Recruiting
		+Nivolumab (anti-PD1) and Ipilimumab (anti-CTLA4) (group A) + Nivolumab (anti-PD1) + lirilumab (anti-KIR) (group B)	I/II	Advanced solid tumors	No standard therapy available (phase I) ≥ 1 prior line (phase II)	NCT03347123	Active, not recruiting
		+ Durvalumab (anti-PDL1)	I/II	Advanced solid tumors	≥ 1 prior line	NCT02318277	Active, not recruiting
		+ Pembrolizumab (anti-PD1) And mFOLFOX6 Or (anti-PD1) Gemcitabine and Nab-Paclitaxel Or Carboplatin and Paclitaxel Or Pemetrexed, and Platinium agent Or Cyclophosphamide Or Gemcitabine and Platinium agent Or Platinium agent and 5-Fu	I/II	Advanced solid tumors	Not specified	NCT03085914	Active, not recruiting
		+/− Pembrolizumab (anti-PD1) Or +/−Pembrolizumab (anti-PD1) and Carboplatin or Cisplatin and Paclitaxel Or +/− Pembrolizumab (anti-PD1) and Carboplatin and Paclitaxel	I	Advanced solid tumors (Japanese population)	No standard therapy available	NCT02862457	Active, not recruiting Preliminary safety and efficacy results published [294]
		+ Pembrolizumab (anti-PD1) and Azacitidine (DNA methyl transferase inhibitor) Or + INCB057643 (BET inhibitor) + Pembrolizumab (anti-PD1) Or + INCB059872 (LSD1 inhibitor) and Pembrolizumab (anti-PD1)	I/II	Advanced solid tumors	No standard therapy available	NCT02959437	Active, not recruiting
		No	I	Advanced solid tumors	No standard therapy available	NCT01195311	Completed, safety results published [295]
		+ Pembrolizumab (anti-PD1) and INCAGN01876 (anti-GITR)	I/II	Advanced solid tumors	No standard therapy avaialble	NCT03277352	Active, not recruiting
		+ Itacitinib (JAK inhibitor)	I	Advanced solid tumors including TNBC	No standard therapy available	NCT02559492	Active, not recruiting
		No	Ib	Resectable metastatic solid tumors	Eligible for surgical resection and no standard therapy available	NCT03471286	Recruiting
	GDC-0919 (navoximod)	+ Atezolizumab (anti-PD-1)	Ib	Advanced solid tumors	≥ 1 prior line	NCT02471846	Active, not recruiting
	NLG802	No	I	Advanced solid tumors	Not specified	NCT03164603	Recruiting

**Abbreviations:** TAM, tumor-associated macrophages; CSF-1R, colony-stimulating factor 1 receptor; CSF-1, colony-stimulating factor 1; PD-1, programmed death 1; PD-L1, programmed death ligand 1; SBRT, stereotactic body radiation therapy; CCL2, C-C Motif Chemokine Ligand 2; CCR2, C-C Motif Chemokine Receptor 2; CD47, cluster of differentiation 47; SIRPα, signal regulatory protein alpha; IDO, Indoleamine 2,3-dioxygenase; TLR7, toll-like receptor 7; NK-cells, natural-killer cells; CD40, cluster of differentiation 40; CD94, cluster of differentiation 94; KIR, Killer Immunoglobin Receptors; NKG2A, NK group member 2A; CTLA-4, cytotoxic T-lymphocyte-associated protein 4; KIR2DL1, Killer cell immunoglobulin-like receptor 2DL1; JAK, janus kinase; mTOR: mammalian target of rapamycin; BC, breast cancer; TNBC, triple-negative breast cancer; LSD1, lysine specific demethylase 1; BET, Bromodomain and Extra-Terminal motif; EGFR, epidermal growth factor receptor; VEGF-A, vascular endothelial growth factor A.

**Table 5:** Overview of immune-related markers’ characteristics including origin of expression and their role in anti-tumor immunity.

Marker	Types of Cells Expressed	Function on Anti-tumor Immunity
LAG-3	Effector T-cells, Tregs, NK-cells, B-cells, dendritic cells (DC)	Co-inhibitory
TIM-3	CD8+, CD4+ T helper 1 cells (Th1 cells), Tregs, NK cells, DC, monocytes, macrophages	Co-inhibitory
VISTA	CD8+, CD4+ T-cells, Tregs, NK cells, DC, monocytes, macrophages, granulocytes	Co-inhibitory
TIGIT	Effector, memory, follicular helper (Tfh) T-cells, Tregs, NK-cells	Co-inhibitory
GITR	T-cells	Co-stimulatory
B7-H3	T-cells, antigen-presenting cells (APC), NK-cells	Co-stimulatory Co-inhibitory
ICOS	T-cells	Co-stimulatory Co-inhibitory
4-1BB	Effector, helper T-cells, Tregs, B-cells, NK-cells, DC, neutrophils, eosinophils, mast cells, monocytes, macrophages	Co-stimulatory
CD27	T-cells, B-cells, NK-cells	Co-stimulatory
OX40	Tregs, neutrophils, NK-cells and NKT-cells, CD4+ and CD8+ T-cells (upon TCR stimulation)	Co-stimulatory
BTLA	T-cells, B-cells, Tfh cells, macrophages, DC, NKT-cells, NK-cells	Co-inhibitory
A2aR	T-cells, NKT-cells, B-cells, monocytes, macrophages, DC, NK-cells, mast cells, eosinophils, platelets	Co-inhibitory
CD73	B-cells, CD8+, CD4+ T-cells, Tregs, neutrophils, MDSC, monocytes, macrophages, DC, NK-cells, endothelial cells, cancer cells	Co-inhibitory
CD39	Platelets, endothelial cells, cancer cells	Co-inhibitory
CCR2	Monocytes, macrophages	Co-inhibitory
CD47	Cancer cells	Co-inhibitory
CD40	APC, macrophages	Co-stimulatory
CD94/NKG2A	NK-cells, CD8+ T-cells	Co-inhibitory
NKG2D	NK-cells	Co-stimulatory
IDO	Cancer cells, stromal dendritic-like cells, myoepithelial cells	Co-inhibitory
